# Knockdown of Ki-67 by Dicer-Substrate Small Interfering RNA Sensitizes Bladder Cancer Cells to Curcumin-Induced Tumor Inhibition

**DOI:** 10.1371/journal.pone.0048567

**Published:** 2012-11-12

**Authors:** Sivakamasundari Pichu, Swapna Krishnamoorthy, Andrei Shishkov, Bi Zhang, Peter McCue, Biddanda C. Ponnappa

**Affiliations:** Department of Pathology, Anatomy and Cell Biology, Thomas Jefferson University, Philadelphia, Pennsylvania, United States of America; The University of Texas M. D. Anderson Cancer Center, United States of America

## Abstract

Transitional cell carcinoma (TCC) of the urinary bladder is the most common cancer of the urinary tract. Most of the TCC cases are of the superficial type and are treated with transurethral resection (TUR). However, the recurrence rate is high and the current treatments have the drawback of inducing strong systemic toxicity or cause painful cystitis. Therefore, it would be of therapeutic value to develop novel concepts and identify novel drugs for the treatment of bladder cancer. Ki-67 is a large nucleolar phosphoprotein whose expression is tightly linked to cell proliferation, and curcumin, a phytochemical derived from the rhizome *Curcuma longa,* has been shown to possess powerful anticancer properties. In this study, we evaluated the combined efficacy of curcumin and a siRNA against Ki-67 mRNA (Ki-67-7) in rat (AY-27) and human (T-24) bladder cancer cells. The anticancer effects were assessed by the determination of cell viability, apoptosis and cell cycle analysis. Ki-67-7 (10 nM) and curcumin (10 µM), when treated independently, were moderately effective. However, in their combined presence, proliferation of bladder cancer cells was profoundly (>85%) inhibited; the rate of apoptosis in the combined presence of curcumin and Ki-67-7 (36%) was greater than that due to Ki-67-7 (14%) or curcumin (13%) alone. A similar synergy between curcumin and Ki-67-7 in inducing cell cycle arrest was also observed. Western blot analysis suggested that pretreatment with Ki-67-7 sensitized bladder cancer cells to curcumin-mediated apoptosis and cell cycle arrest by p53- and p21-independent mechanisms. These data suggest that a combination of anti-Ki-67 siRNA and curcumin could be a viable treatment against the proliferation of bladder cancer cells.

## Introduction

Bladder cancer is the most common urological cancer in Southeast Asia and the second most frequent urological malignancy in North America [1]. Transitional cell carcinoma (TCC) accounts for greater than 90% of patients diagnosed with bladder cancer [2]. Greater than 70% of the TCC tumors are superficial tumors confined to bladder mucosa and lamina propia -Ta or T1 staged tumors and the remaining are of the invasive type. The incidence of urinary bladder cancer has continuously increased during the past two decades [3]. A preferred treatment for superficial tumors is transurethral resection (TUR), but the risk of recurrence (60–70%) due to the reattachment of released tumor cells and death from the disease (10–30%) is high [4]. The primary approach to prevent the recurrence of the tumor, after TUR, has been intravesical instillation therapy (IVI) using chemotherapeutic drugs [5], [6] but the cytotoxic effects of the drugs are of concern. Because of its higher efficacy, intravesical immunotherapy using *Bacillus Calmette Guerin* (BCG) has become the treatment of choice during the past three decades. However, induction of cystitis and systemic toxicity are some of its serious side effects [2]. Despite the aggressive therapies, patients with bladder cancer have a 5-year survival rate of only about 50% [7]. Further, substantial numbers of bladder cancer patients are resistant to conventional intravesical therapy and therefore, it is necessary to develop newer and preferably less toxic approaches to combat the disease.

One of the newer approaches to suppress tumor progression is by using gene-specific drugs such as, antisense oligonucleotides (AsODNs) or small interfering RNAs (siRNAs) against mRNAs of tumor-specific proteins. Following the discovery of RNA interference (RNAi) in a variety of species [8]–[10], there has been a tremendous interest in harnessing the therapeutic potential of siRNAs in the treatment of various diseases [11], including cancer [12] and inflammatory diseases [13]. Ki-67 is a large nucleolar phosphoprotein whose expression is tightly linked with the cell cycle and it is strictly associated with cell proliferation [14]. It is a DNA-binding protein with a primary role in maintaining higher order structure for DNA during the process of mitosis. Detailed cell cycle analysis revealed that the Ki-67 antigen is present in nuclei of proliferating (G1-, S-, G2- phase and mitosis) but not in the nuclei of quiescent or resting cells (G0- phase) [15]. This suggests that Ki-67 inhibitors may have relative specificity for malignant cells. Yoa et al., [16] reported that among many of the cancer-related genes tested, that of Ki-67 expression was one of the highest in rat bladder tumors, reaching nearly 20-fold higher levels compared to normal tissue. Thus, Ki-67 has been one of the genes of interest to target, using AsODNs [17], [18] or siRNAs [19], [20]. In more recent report, AsODN against Ki-67 was used in Phase-I clinical trials for the treatment of human bladder cancer [21]. Although, the efficacy of AsODNs demonstrate the proof of the principle, it is known that siRNAs are at least an order of magnitude more sensitive than AsODNs [22], [23] and hence, much lower amount of the drug need to be used for similar efficacy. Further, it has been shown that the longer (27 bp) Dicer-substrate siRNAs (DsiRNAs) are more sensitive than the standard 19-21 bp siRNAs [24]. Thus, siRNAs/DsiRNAs provide a better tool than AsDONs to target tumor-specific genes.

In addition to the antisense molecules, in recent years, drugs of plant origin have also received much attention due to their enormous potential in the prevention and treatment of cancer [25]. Curcumin is a polyphenolic phytochemical derived from the rhizome, *Curcuma longa*. Because of its powerful antiproliferative and anti-inflammatory effects, it has drawn a lot of attention from researchers in the cancer field. To date, there are more than 70 clinical trials at various stages of completion, testing the efficacy of curcumin against many diseases (www.clinicaltrials.gov). The role of curcumin in modern medicine has been recently reviewed [26]–[28]. The anticancer potential of curcumin is derived from its ability to suppress proliferation of a wide variety of tumor cells, by down-regulating the expression of proliferating genes such as COX2, MMP-9, chemokines, cyclin D1 and the nuclear factor κB (NF-κB) [29]. Tharakan et al., [30] using an orthotopic mouse model of bladder cancer, reported that curcumin abolished the constitutive activation of NF-κB in the tumor tissue, induced apoptosis and decreased COX-2 expression. However, in the presence of the chemotherapeutic drug, gemcitabine, low concentrations of curcumin potentiates the effects of the drug through the modulation of the NF-κB signaling pathway [31]. These observations clearly demonstrate the therapeutic potential of curcumin in the treatment of cancer.

Since curcumin inhibits the proliferation of tumor cells by targeting multiple sites in the apoptotic and proliferation pathways, we hypothesized that superimposition of the multi-targeted curcumin following molecular inhibition of Ki-67, would have a greater inhibitory effect on tumor growth. Indeed, our data show, for the first time, that pretreatment of bladder cancer cells with DsiRNA against Ki-67 mRNA promotes cell-cycle arrest and sensitizes the cells to curcumin-induced apoptosis. The putative molecular targets of curcumin and Ki-67 DsiRNA are discussed.

## Materials and Methods

### SiRNA Design

A total of three DsiRNA duplexes targeted against rat Ki-67 mRNA were designed (Integrated DNA Technology, Coralville, IA, USA). The sequences of the three DsiRNAs are as follows; **1) Ki-67 -2;** Sense sequence 5′ – CGA ACA AAG UCA UCA GAC ACA GUG A-3′; antisense sequence 5′- UCA CUG UGU CUG AUG ACU UUG UUC GUU-3′; **2)** **Ki-67-7**; Sense sequence 5′ – CAA GAG UGA GGG AGU GCC UUU GAA G-3′; antisense sequence 5′ – CUU CAA AGG CAC UCC CUC ACU CUU GUU -3′ and **3)** **Ki-67-9;** Sense sequence 5′- CCA CCA GAG CCA AUA GAU ACU UCA G-3′ and antisense sequence 5′- CUG AAG UAU CUA UUG GCU CUG GUG GUG-3′. An irrelevant DsiRNA, hereafter referred as ‘control’ DsiRNA was designed and has following sequence; Sense sequence 5′- CAA GAG UGA GGG AGU GCC UUU GAA G-3′; antisense sequence 5′ – CUU CAA AGG CAC CGG AUC ACU CUU GUU-3′. Although the abbreviation “DsiRNA” is initially used to emphasize the fact that the longer siRNAs were used in the current study, the generic term “siRNA” is used interchangeably throughout the text.

### Cell Culture

Transplantable rat-derived TCC cells (AY-27 cells) were kindly provided by Dr. Ronal Moore (University of Alberta, Canada). The characterization of a bladder tumor model using this transplantable cell line has been reported [32]. The cells were maintained in monolayers, in a culture medium consisting of RPMI-1640 (Gibco-BRL), supplemented with 10% FBS, 30 mM HEPES (pH 7.3), 1 mM pyruvate, penicillin-streptomycin and 2.0 g/l of NaHCO_3_. Cells were maintained at 37°C in 5% CO_2_ and 95% air atmosphere by the method of Xiao *et al* [32]. AY-27 cells were passaged when confluent by standard trypsinization and reculturing procedure. Where indicated, for comparative purposes, human-derived T-24 bladder cancer cell lines (American Type Culture Collection) were also used in this study and maintained in tissue culture as described for AY-27 cells.

### SiRNA Transfection

The day before transfection, cells were trypsinized, diluted with fresh medium and transferred to 12-well plates (0.8–1.0×10^5^cells/well). DsiRNAs were transfected using specifically designed reagent for siRNA transfection (Lipofectamine™ RNAiMax) according to manufacturer’s protocol (Invitrogen, USA). Routinely, transfection was carried out at 8–10% cell confluency.

### Curcumin Treatment

When indicated, tumor cells were treated with various concentrations of curcumin (Sigma-Aldrich, USA), either alone or in combination with DsiRNA. Curcumin was dissolved in DMSO, and in any given experiment, the final concentration of DMSO was always less than 0.1% by volume.

### Cellular Growth Curve

For evaluating tumor proliferation, tumor growth was assessed by measuring the total number of cells at various stages. At each stage, cells were detached by trypsinization, washed with PBS, stained with trypan blue and the live cells were counted using a hemocytometer. Each experimental condition was repeated three to six times.

### MTT Assay

The antiproliferative effects of siRNA against Ki-67 in the presence or absence of curcumin was determined by the MTT assay. The assay was based on the ability of mitochondria to reduce 3-(4, 5 dimethylthiaol-2-yl)-2, 5-diphenyltetrazolium bromide (MTT) dye in rat (AY-27) and human (T-24) bladder cancer cells as detailed by Plumb [33]. Briefly, following exposure of the cells to various treatment conditions, medium was removed, rinsed with PBS, and MTT solution (5 mg/ml in RPMI medium) was added and incubated for 37°C for 3 hours. Subsequently, the medium was removed and replaced with an acid-isopropanol solvent to dissolve the precipitate. The contents were placed on a shaker for 5 mins and absorbance measured at 570 nm.

### Quantitative Real-time PCR (qPCR)

After transfection with siRNAs and/or treatment with curcumin, cells were harvested at the appropriate interval and total RNA was prepared by PureLink RNA mini kit (Invitrogen, USA). For cDNA synthesis, 1 µg of total RNA was reverse transcribed to cDNA in 20 µl reactions using Quantitect Reverse transcription kit (Invitrogen, USA). qPCR was performed with the Light cycler® 480 detection system (Roche, USA) using the Brilliant® SYBR® Green QPCR Master mix protocol (Stratagene, USA). Briefly, 2 µl of cDNA was amplified by PCR in 25 µl reactions containing 12.5 µl of 2× SYBR green reagents and 0.2 µM of each of the primers. The initial incubation for 10 min at 95°C was followed by 40 cycles at 95°C for 15 sec and 60°C for 1 min. Each experiment was carried in triplicate.

### Ki-67 Protein Expression: Immunofluorescence Staining

Cells were cultured on 25 mm glass coverslips in 6-well plates. Following exposure to siRNA and/or curcumin under various conditions, cells were washed with cold PBS and fixed with 4% formaldehyde. After washing with PBS, the cells were incubated with Ki-67 primary antibody (M19, Santa Cruz Biotechnology Inc.,) (1∶100) for 3 hours and washed thrice with PBS. The coverslips were then incubated with FITC-labeled secondary antibody (1∶400) in the dark for an hour at room temperature, washed with PBS and stained for 10 min with propidium iodide (PI) solution. The coverslips were then washed with PBS and mounted on a slide in the presence of mounting medium (Antifade solution, Invitrogen, USA). The slides were then viewed under Bio Rad Radiance 2001 system coupled to an Olympus IX70 microscope with 60× and 40× oil immersion objectives (UAp0340, NA 1.35). A dual line Kr/Ar ion laser source was used for imaging with excitation settings of 488 nm for FITC and 588 nm for PI.

### Detection of Apoptosis by Annexin V Assay

Apoptosis was measured using PE Annexin V Apoptosis Detection Kit I (BD Biosciences, USA) as per manufacturer’s protocol. Briefly, following exposure to siRNA with or with out curcumin, the cells were washed with cold PBS and then resuspended in binding buffer followed by the addition of PE Annexin V and 7-Amino-Acitomycin (7-AAD) and analyzed in Beckman Coulter’s Epics XL-MCL™ Flow Cytometer, (USA). Routinely, the average fluorescence derived from 20,000 cell counts per sample was recorded.

### Cell Cycle Analysis

To determine the effect of anti Ki-67 DsiRNA and/or curcumin on the cell cycle phases, AY-27 cells were exposed to various experimental conditions, the media removed and the cells were detached by trypsinization. The cells were then washed with PBS and fixed in ice-cold 70% ethanol for 2 hours at −20°C. The cells were then washed once with PBS, resuspended in 300 µl of an ice-cold modified PI solution (50 µg/ml PI solution, 0.1% Triton X-100, and 0.1% sodium citrate, in PBS) and incubated for 30 min before analysis. PI fluorescence was measured by Beckman Coulter’s Epics XL-MCL™ Flow Cytometer, (USA). Routinely, the average fluorescence derived from 20,000 cell counts per sample was recorded. Cells were scored as percentage of cells in each of the cell cycle phases (G_o_/G_1,_ S and G2/M).

### Western Blot Analysis

Bladder cancer cells, after exposure to curcumin and DsiRNA under various conditions, were trypsinized, washed with PBS, and lysed with ice-cold RIPA (Sigma- Cat # R0278) buffer. The lysates were kept on ice for 20 min, vortexed 3–4 times, and then centrifuged in a microfuge at 12,000 rpm for 15 min at 0–4°C. The supernatants were removed and assayed for protein content. Aliquots (40 µg) of protein from each sample were dissolved in 4× NuPage lithium-dodecyl sulfate (LDS) and subjected to SDS-PAGE under reducing conditions. The separated proteins were transferred onto a nitrocellulose membrane and blocked in either 5% bovine serum albumin (BSA) or 5% non-fat dry milk in a Tris-buffered saline containing 0.1% Tween-20 (TBST) by standard procedures. The membranes were then incubated overnight at 4°C with the primary antibodies against following antigens: β-actin, Cyclin E, and p53 (Santa Cruz Biotechnology, Inc., Santa Cruz, CA, USA). Other antibodies used were against cleaved Caspase3, phosphorylated-RB (pRb-P) (Cell Signaling Technology, Inc, Danvers, MA ), Cyclin D1, (BD Bioscience, San Jose, CA), Ki-67, NF-κB (p65 subunit), p27/Kip and p21 (Abcam, Cambridge, MA). The blots were then washed extensively with TBS-T and incubated with appropriate horseradish peroxidase-conjugated anti-mouse or anti-rabbit (Pierce Biotechnology/Thermo Fisher Scientific, Rockford, IL) secondary antibodies at dilutions 1∶15,000 and 1∶20,000 respectively for 2 h at RT before the final wash with TBST. The protein bands were detected by enhanced chemiluminescence system using SuperSignal West Pico Chemiluminescent Substrate (Thermo Fisher Scientific, Rockford, IL). The KODAK Image Station 440CF (Kodak Scientific Imaging Systems, New Haven, CT) was used to visualize and quantify the signal net intensity of bands. All experiments were performed in triplicates. Representative blots are shown.

### Protein Assay

Protein content in cell lysates were measured using Micro BCA™ protein assay kit (Pierce Biotechnology/Thermo Fisher Scientific, Rockford, IL) using bovine serum albumin as the internal standard.

### Statistical Analysis

Where indicated, values are presented as mean ± S.E of (n) determinations. Data were subject to statistical analysis by paired student’s t-test using the Microsoft Excel program and the values with P<0.05 were considered to be statistically significant.

## Results

### Inhibition of Ki-67 Gene Expression and Cell Proliferation by DsiRNA

**Figure 1:**
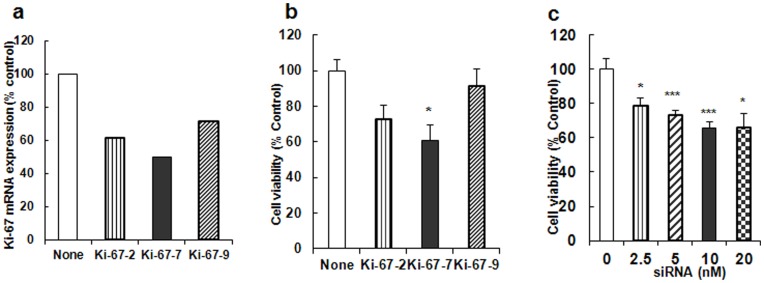
Effect of various DsiRNA constructs on a) Ki-67 mRNA expression and b) cell proliferation in bladder cancer cells; c) Dose-response studies with Ki-67-7. a) Rat TCC cells (AY-27) were cultured in 12-well plates (∼0.8 ×**10^5^ cells/well) as described in Materials and Methods.** After 24 h, they were transfected with each of the siRNA constructs (Ki-67-2, Ki-67-7 and Ki-67-9) targeted against Ki-67 mRNA. After 48 h, RNA was extracted and the levels of mRNA were determined by quantitative PCR as described in Methods. Cells treated with the transfection reagent alone were used as the control. The rate of expression of Ki-67 mRNA was normalized to the expression of β-actin. Values represent data from one (of two) experiment using the average of triplicate determinations. b) AY-27 cells were cultured in 12-well (0.8×10^5^ cells/well) plates for 24 h and transfected with various siRNA constructs as described in Methods. Cell viability was determined 48 h after transfection by MTT assay. Cells treated with transfection reagent alone served as the control ( = 100%). Bars indicate values which are the means ± S.E of three determinations. (^*^P<0.05). c) AY-27 cells were cultured in 12-well plates (0.8×10^5^ cells/well) as described in Methods. Forty eight hours after siRNA transfection, the effect of various concentrations of Ki-67-7 on cell viability was determined by MTT assay. Cells treated with transfection reagent alone served as the control. Cell viability values are expressed as percent control and are the means ± S.E of 3-8 determinations (^*^P<0.05, ^***^P<0.005).

The *in vitro* efficacies of three DsiRNA constructs targeted to rat Ki-67 mRNA were tested as described above in AY-27 cells. As shown in Figure 1A, out of the three DsiRNAs (Ki-67-2, -7, -9), Ki-67-7 showed maximum inhibition. The mRNA expression, analyzed by qPCR, showed a 39%, 50% and 28% decrease in the expression of Ki-67 mRNA by Ki-67-2, Ki-67-7 and Ki-67-9 respectively when compared to cells treated with transfection reagent alone. Similarly, cell viability assay by MTT showed that Ki-67-7 treatment was the most effective of the three (Fig. 1B). Thus, Ki-67-7 was chosen as the most effective DsiRNA against Ki-67 mRNA for subsequent studies. Further, dose-response studies showed that Ki-67-7 was most effective at 10 nM concentration (1c). Under our experimental conditions, a further increase in Ki-67-7 concentration did not enhance the anti-proliferative effect of the siRNA.

### Effect of Curcumin on Tumor Proliferation: Synergy with Ki-67-7

**Figure 2:**
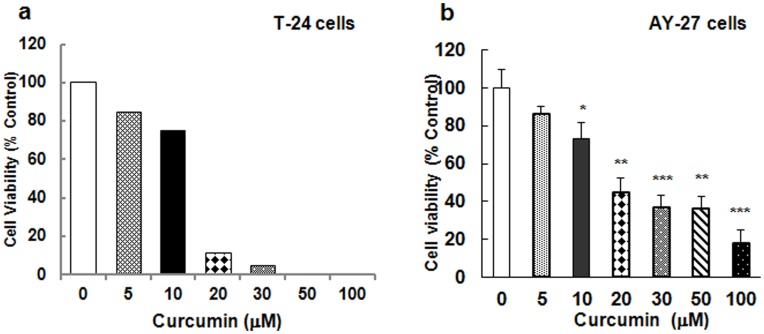
Dose-dependent effects of curcumin on bladder cancer cell proliferation. T-24 (2a) and AY-27 (2b) cells were plated in 12-well plates (0.8×10^5^ cells/well) and cultured for 48 hr (40–50% confluence) as described in Methods. Following exposure to various concentrations of curcumin for 24 h, cells were washed with PBS and incubated for another 24 h in curcumin-free medium. Cells incubated with DMSO (0.1%) alone were treated as controls. DMSO alone had little impact on tumor cell growth (data not shown). Cell proliferation was assessed on the basis of cell viability, measured by MTT assay. Cell viability was expressed as percent of viability observed in DMSO-treated control cells. Values are from a representative (of two) (2a) or 3–8 (mean ± S.E) determinations (2b). ^*^P<0.05, ^**^P<0.01,^ ***^P<0.005.

**Figure 3:**
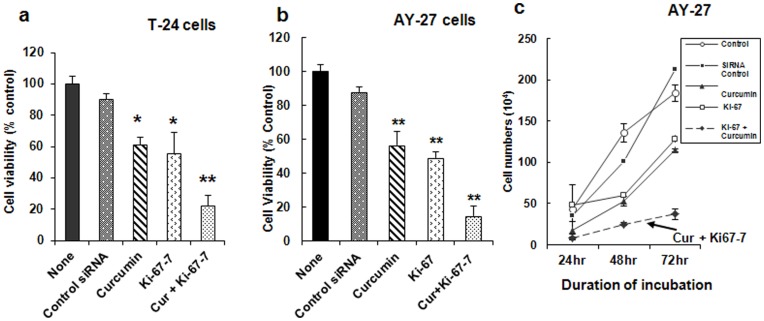
Effect of the combined treatment of anti-Ki-67 DsiRNA and curcumin on bladder cancer cells. Equal number (0.8×10^5^ cells/well) of bladder cancer cells T-24 **(3a)** and AY-27 **(3b)** cultured as described in Methods were either transfected with Ki-67–7 or control DsiRNA (10 nM) or treated with the transfection reagent alone for 24 h. Subsequently, where indicated, curcumin (10 µM) was added to the cells and incubated for 24 h followed by an additional 24 h incubation in the absence of curcumin. Cell viability was determined by MTT assay at the end of 72 h from transfection. Cells treated with DMSO plus transfection reagent served as the control. Data presented are the mean ± S.E of 3–5 determinations (^*^P<0.05, ^**^P<0.01). **3c)** AY-27 cells (0.8×10^5^ cells/well) were treated exactly as described above in legend to Figures 3A and 3B and at the end of 24, 48 and 72 h after curcumin treatment (which is same as 48, 72 and 96 h after siRNA transfection), cell growth was assessed based on cell numbers as described in Methods. Data are mean ± S.E of three determinations.

The effects of curcumin and Ki-67-7 were tested in both human-derived (T-24) and rat-derived (AY-27) bladder cancer cells. Sequence comparisons showed that there was 80% homology in the target sequence of Ki-67-7 between rat and human Ki-67 mRNAs. In dose-response studies, we observed that tumor cell growth was inhibited by curcumin in a dose-dependent way (Figs. 2A and 2B) in both cell types but T-24 cells were more sensitive to curcumin than AY-27 cells at 20 µM and above. However, at 10 µ M, the degree of inhibition (25–30%) was similar in both cell types. In both cases, a drastic reduction (60–85%) in cell viability was observed between 10 and 20 µM curcumin. However, for purposes of comparing the combinatorial effects of DsiRNA and curcumin (CusiRNA), the lower dose (10 µM) of curcumin was used along with the optimal dose (10 nM) of the DsiRNA (Ki-67-7). Data showed almost complete (85%) inhibition of cell viability during the combined treatment of curcumin and DsiRNA (Fig. 3A and 3B). Control siRNA had insignificant effect on cell viability suggesting the specificity of Ki-67-7 against its target.

The antiproliferative effect was also determined by monitoring the cell growth curve. The growth curves based on cell numbers were determined at 24, 48 and 72 hours after DsiRNA and curcumin treatment. Combined treatment (CusiRNA) resulted in a marked inhibition of cell proliferation and mimicked the same trend as that of cell viability studies (Fig. 3C). The cellular protein values also correlated well with growth curves (data not shown).

### Effects of Ki-67-7 and Curcumin on Ki-67 mRNA and Protein Expression

**Figure 4:**
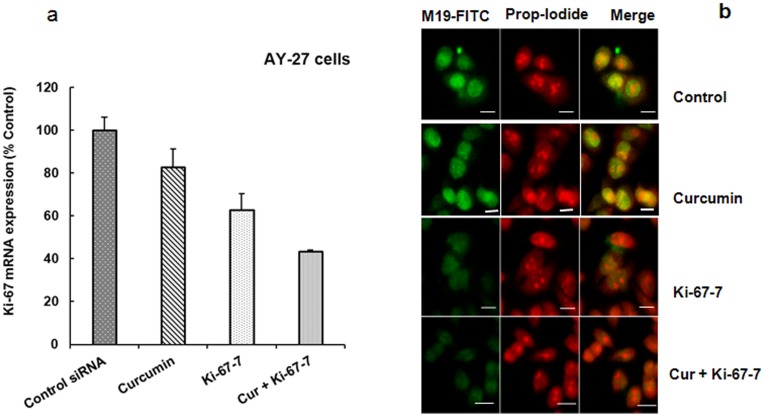
Effect of Ki-67-7 and curcumin on Ki-67 a) gene and b) protein expression in bladder cancer cells. a) AY-27 cells were treated with Ki-67 siRNA in the presence or absence of curcumin exactly as described in legend to Figures 3A and 3B. At the end of the treatment, RNA was extracted and Ki-67 mRNA expression was determined by quantitative PCR using β-actin as the internal standard as described in Methods. Ki-67 mRNA levels are expressed as percent of the rate of expression in control cells which were treated with transfection reagent and DMSO. Bars indicate values which are the mean ± S.E of three determinations. b) AY-27 cells were grown on glass coverslips placed inside 12-well plates and treated with siRNA (Ki-67-7), curcumin or as a combination, as described in the legend to Figures 3A and 3B. At the end of the treatment period, cells were exposed to Ki67 primary antibody (M19) and FITC-labeled secondary antibody followed by PI (Propidium iodide), and observed under confocal microscopy as described in Methods. Scale bar inserts  = 5 µm. Increased down-regulation of Ki-67 protein due to Ki-67-7 was observed.

To determine whether the reduction in tumor cell viability during CusiRNA treatment was related to Ki-67 protein levels, we measured the levels of both Ki-67 mRNA and protein expression. Data show reductions in the levels of Ki-67 mRNA by 17%, 37% and 57% when exposed to curcumin, Ki-67-7 and CusiRNA respectively (Fig. 4A). Similarly, using immunofluorescence staining technique, we observed that unlike Ki-67-7, curcumin *per se* had minimal impact on Ki-67 protein expression (Fig. 4B).

### Induction of Apoptosis by Ki-67-7 and Curcumin

**Figure 5:**
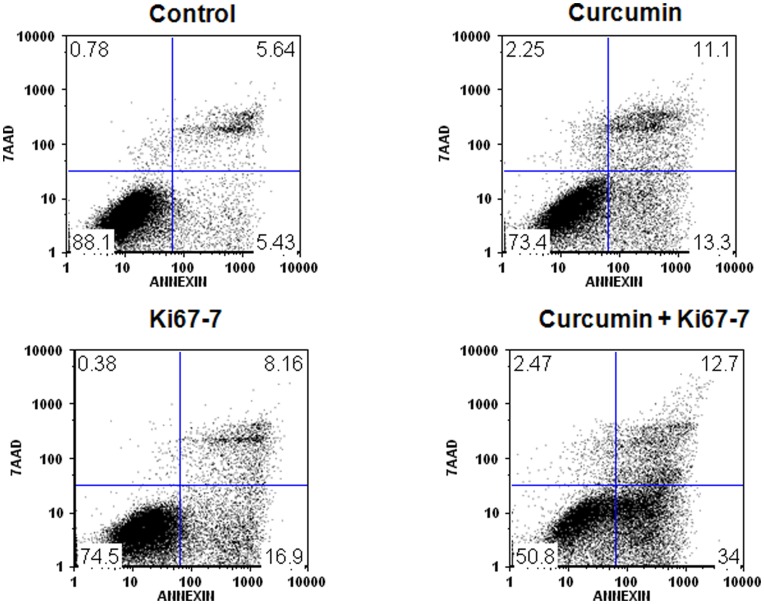
Determination of Ki-67-7 and curcumin-induced apoptosis by flow cytometry. AY-27 cells were treated with anti-Ki-67 siRNA, curcumin or in the combined presence of both as described in legend to Figures 3A and 3B. At the end of the treatment period, the cells were harvested, stained with fluorescence-conjugated Annexin V and 7AAD as described in Methods. Following flow cytometric analysis, the percentage (number inserts in the figure) of normal cells or those undergoing apoptosis and necrosis was calculated using appropriate software (Flow Jo v.9). The rate of apoptosis was measured by adding the percentages in the right two quadrants in each of the figures. The data are from a representative (of two identical) experiment.

Loss of cell viability is often the result of apoptosis or necrosis. The extent of apoptosis, an early indicator of cell death, was quantified by flow cytometric analysis using Annexin V to detect the various stages of apoptosis (Fig. 5). Data suggest mild induction of apoptosis when the cells were separately treated with curcumin or Ki-67-7. However, the extent of apoptosis (the right two quadrants in Fig. 5) in the presence of CusiRNA was found to be synergistic (36% vs 13% for curcumin and 14% for Ki-67-7) after correcting for baseline apoptosis (11%) in control cells (Fig. 5). The figure also indicates (left top quadrant in Fig. 5) that the extent of cell necrosis (Annexin V negative, 7AAD positive) was minimal during all of the treatment conditions.

### Ki-67-7 and Curcumin on Cell Cycle Phases

**Figure 6:**
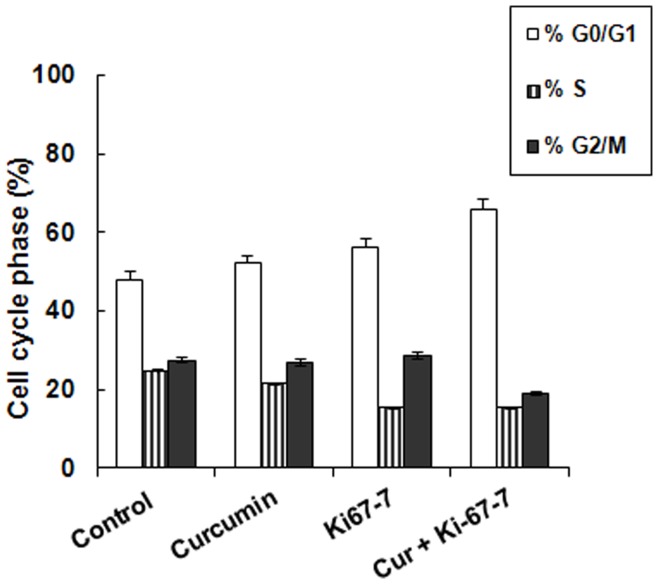
Effect of Ki-67-7 and curcumin on cell cycle phases. AY-27 cells were treated with Ki-67-7, curcumin or with both, as described earlier in legend to Figures 3A and 3B. At the end of the incubation period, the cells were treated with PI and the distribution of cells in various cell cycle phases were determined by flow cytometry as described in Methods. Cells were scored as percentage distribution of cells in each of the cell cycle phases (G_o_/G_1,_ S and G2/M). Bars indicate values which are the means ± S.E of three determinations.

The effect of Ki-67-7 on cell cycle was determined in the presence or absence of curcumin. Data show a general shift in the direction of G0/G1 phase in all of the treatment conditions, but the effect was maximal in the presence of CusiRNA (Fig. 6). There was no significant change in G2/M phase when separately exposed to either curcumin or Ki-67-7, but a 33% decrease was observed when the cells were exposed to CusiRNA. Interestingly, with an increase in G0/G1 and decrease in G2/M phases, there was a two-fold shift (ratio of G0/G1 to G2/M) towards growth arrest in the presence of CusiRNA compared to control (Fig. 6).

### Effect of Ki-67-7 and Curcumin on Multiple Signaling: Molecular Targets in Cell Cycle And Apoptosis

**Figure 7:**
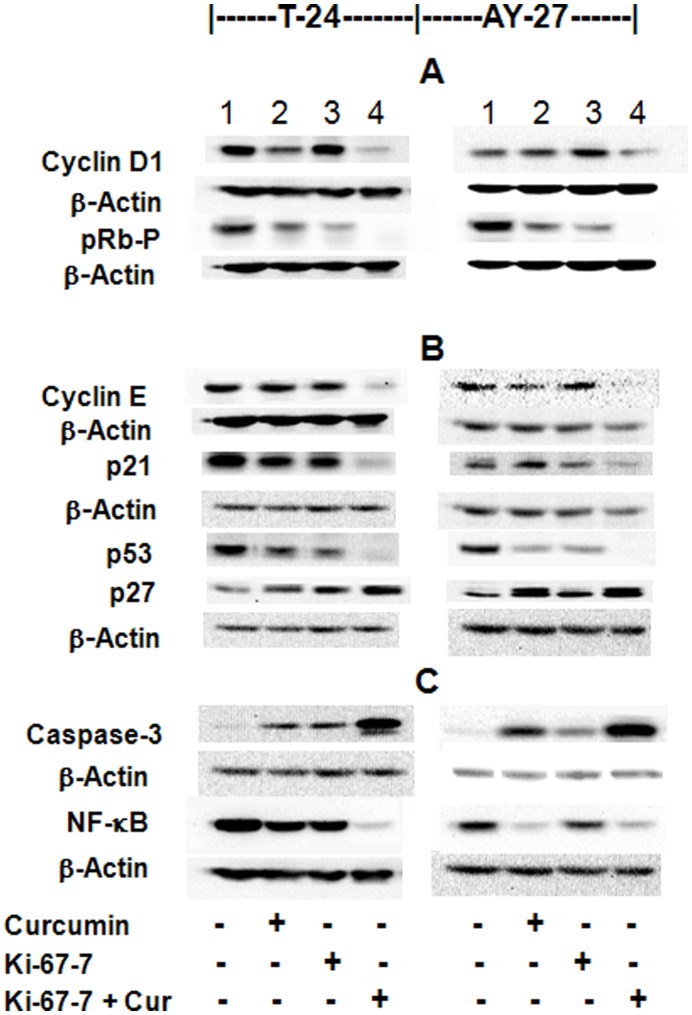
Effect of Ki-67-7 and curcumin on the regulatory proteins of cell cycle phases and apoptosis: Western blot analysis. Bladder cancer cells (T-24 and AY-27) were cultured and exposed to Ki-67-7 and curcumin as described above in legend to Figures 3A and 3B. At the end of the treatment period, total protein was extracted and subjected to Western blotting using appropriate antibodies as described in Methods. For purposes of associating proteins with specific roles, they were grouped in separate categories, such as gene transcription (A), cell-cycle progression (B) and apoptosis (C). β-actin was used as the internal control. Since different proteins (e.g. Cyclin D1 and NF-κB) from the same membrane were identified in separate categories, the same β-actin blot is displayed on more than one occasion. Data presented are western blots from a single experiment, which is representative of 2–3 identical experiments.

Inhibition of cell cycle progression, induction of apoptosis or a combination of both, are the key events associated with tumor inhibition. Data presented above clearly suggest that a combination of curcumin and Ki-67-7 affect both the events. In order to understand the role of some of molecular intermediates that are likely to affect these events, we determined the levels of some of the regulatory proteins associated with apoptosis and cell cycle progression. Increased levels of cleaved-caspase3 by Western blot analysis confirmed the induction of apoptosis, which was most pronounced in the CusiRNA group (Fig. 7). However, there was a marked reduction in the levels of the tumor suppressor protein, p53, in the same group. Cyclins D1 and E, the proteins associated with G1-S transition in the cell cycle phases, were also profoundly inhibited by CusiRNA. As expected, reduction in cyclin D1 was associated with a reduction in the levels of pRb-P. Reduction in p53 was also associated with a reduction in p21, an inhibitor of cyclin E. However, it was the increase in p27 protein, another inhibitor of cyclin E that seemed to correlate well with the inhibition of cyclin E in the CusiRNA group. These observations were similar in both cell types. On the other hand, the inhibition of the transcription factor NF-κB was more attributable to curcumin treatment in AY-27 cells whereas in T-24 cells, it was inhibited primarily by CusiRNA.

## Discussion

Transitional cell carcinoma (TCC) of the urinary bladder is the most common cancer in the urinary system. Although the incidence of urinary bladder cancer has continuously increased during the past three decades, there has been little progress in its treatment. Following TUR, tumor recurrence and drug-related toxic side-effects are some of the challenges facing the treatment of bladder cancer. Therefore, the objective of our study was to test a novel approach that was intended to cause significant inhibition of tumor growth with minimal toxicity.

Ki-67 antigen, although often used as a proliferation marker because of its high transcription rate, is beginning to be used as a target in the treatment of various types of cancer [16]–[21]. The role of curcumin in suppressing the pathways linked to oncogenesis, including cell survival, proliferation, invasion and angiogenesis has already been discussed. Effects on multiple sites such as protein kinase C activation, the alteration of transcriptional factors such as, c-jun/AP-2, NF-κB and p53 by curcumin have been reported [34]. However, our studies were aimed at using curcumin as a co-drug since it is now well-established that curcumin can indeed potentiate the effects of anticancer drugs [30]. Therefore, the aim of the present study was to evaluate whether we could demonstrate a synergy between the multi-targeted curcumin and Ki-67-7, the anti-Ki-67 siRNA.

In cell culture studies, it was possible to achieve greater than 50% inhibition of tumor cell growth at 20 µM concentration of curcumin. However, we routinely used a sub-maximal dose of 10 µM, to specifically study the combined interaction of curcumin and anti-Ki-67 siRNA. Our data show that although 10 nM of Ki-67-7 significantly inhibited cell proliferation, almost a total inhibition of bladder cancer cell growth was achieved in the combined presence of curcumin (10 µM) (Fig. 3). The advantage of using low concentrations of two separate classes of drugs can be appreciated when we compare our data with those of Zheng *et al* [19], in which, they had to use a ten-fold higher concentration (100 nM) of anti-Ki-67 siRNA to inhibit tumor proliferation only by 45%. Additionally, with regard to curcumin, a lower dose is preferable since increasing its concentration (>50 µM) is also known to be toxic to non-tumor cells [35].

In the current study, we also attempted to correlate the antiproliferative properties of curcumin and Ki-67-7 on various cellular processes such as apoptosis, cell-cycle progression and Ki-67 expression. Our data show that although curcumin and Ki-67-7 both showed similar degrees of inhibitory effect on cell viability, addition of curcumin to Ki-67-7-treated cells almost completely inhibited cell proliferation with minimal impact on Ki-67 expression (compare Figs. 3B and 4). This suggested that although curcumin *per se* had minimal impact on Ki-67, when Ki-67 expression is suppressed by the siRNA, curcumin-mediated processes operate cooperatively to enhance apoptotic cell death in tumor cells. Thus, our data demonstrate for the first time, a synergy between a low concentration of curcumin and an anti-Ki-67 siRNA (Ki-67-7) in the inhibition of bladder cancer cells. The possible molecular targets of the CusiRNA are discussed below.

The initial testing of the interaction between curcumin and Ki-67-7 against bladder cancer cells was measured by MTT assay. Although MTT assay, which determines cell viability, provides the end result, it does not provide information on the events, such as cell cycle arrest and apoptosis that lead to tumor inhibition. An early indicator of apoptosis is the rapid translocation of phosphatidyl serine from the cytoplasmic side of the plasma membrane to the extracellular surface. The loss of asymmetry can be detected by using the binding properties of Annexin V to cell surface. In the present study, a sensitive assay of apoptosis by flow cytometry using Annexin V, clearly demonstrated the synergy between Ki-67-7 and curcumin during the early stages of apoptosis (Fig. 5). Such an observation is reminiscent of the synergy between curcumin and the chemotherapeutic drugs, such as gemcitabine [31] and paclitaxel [36], except that siRNAs are far less toxic compared to those drugs.

During cell proliferation, the key cell-cycle events involve transition from one phase to the next; resting (G0), increase in cellular contents (G1), DNA replication (S), chromosome check point and preparation for mitosis (G2) and mitosis (M). Since Ki-67 antigen is involved in DNA replication, an inhibition of this protein was expected to affect the cell cycle phases, specifically at the S phase. As expected, there was a 40% reduction in the number of cells in the S phase when treated with Ki-67-7 and marginal reduction by curcumin. However, in the presence of CusiRNA, a major shift towards growth arrest was observed as indicated by a 2-fold increase in the ratio of G0/G1 to G2/M compared to other treatment groups (Fig. 6). Such a drastic shift in cell cycle phases, in favor of growth arrest and against mitosis (G2/M), was associated with an almost total inhibition of tumor cells (Fig. 3). Kausch *et al* [17] have suggested that in the absence of Ki-67, cells that attempt mitosis may be forced into apoptosis. However, our data suggest that the apoptotic process was minimally affected by the inhibition of Ki-67 (caspase-3, Fig. 7) but profoundly enhanced by the addition of curcumin (Fig. 5).

**Figure 8:**
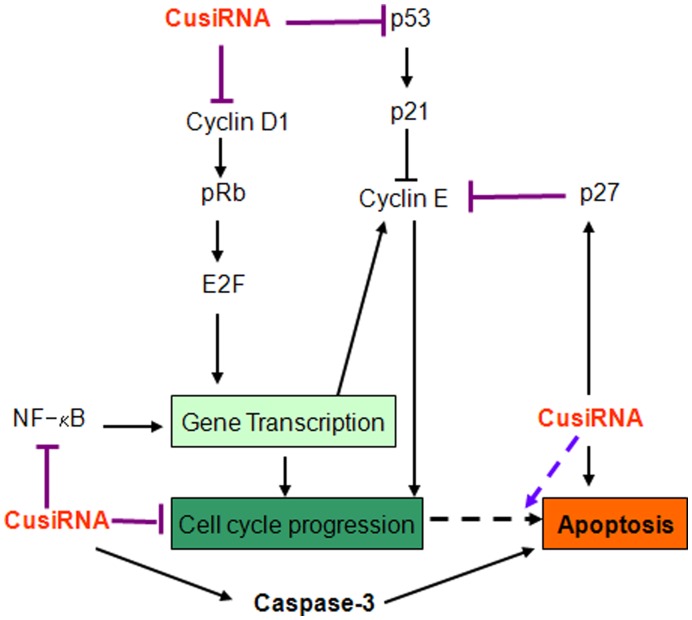
Molecular targets of CusiRNA. The scheme displays the possible molecular targets of Ki-67-7 and curcumin when used in combination (CusiRNA). Arrowheads indicate the activation of indicated proteins; hammer heads indicate inhibition and the broken lines represent the possible role for putative intermediates. Further details are provided in the text.

Although a detailed analysis of the molecular mechanisms underlying the inhibition of tumor cell growth by curcumin and Ki-67-7 is beyond the scope of this study, our data show the following (see Fig. 8 for the scheme): Cyclins D1 and E were both inhibited by CusiRNA, thus affecting the cell cycle progression. Reduction in cyclin D1 was also associated with lower pRb-P levels, a downstream activator molecule regulated by cyclin D1. Further, an increase in the level of p27 by CusiRNA, an inhibitor of cyclin E also favored growth arrest. However, the most striking observation of this study is the profound inhibition of both p53 and p21 proteins by CusiRNA (Fig. 7). These two proteins are generally activated by curcumin treatment [37]. In an earlier study, Tian et al [38] reported that exposure of T-24 cells to curcumin resulted in an increased expression of p53 along with the induction of apoptotic cell death and cell cycle arrest in the G2/M phase. However, unlike in our studies, they had to use a much higher dose (40 µM) of curcumin since the inhibition of tumor growth was low at 10 µM. Since p21 is regulated by p53 [39], the inhibition of p53 may explain the absence of p21, another regulator of cyclin E. Nevertheless, the possibility for tumor cells to undergo apoptosis in the absence of p53 and p21, but in the presence of chemotherapeutic drugs has also been reported [40]. Taken together, our data suggest that in bladder cancer cells, prior exposure to anti-Ki-67 siRNA, predisposes the tumor cells to curcumin-induced growth arrest and apoptosis by non-p53 and non-p21-dependent pathways.

### Conclusions

In summary, our studies show, for the first time, that anti-Ki-67 siRNA, in the combined presence of a low concentration of curcumin, was highly effective against the proliferation of bladder cancer cells. Future studies are required to identify the molecular mechanisms that are involved in the prevention of tumor growth during the combined treatment with anti-Ki-67 siRNA and curcumin. The outcome of our studies could form a new basis for a non-toxic treatment of bladder cancer patients who undergo TUR.
